# F. D. I. International Dental Federation—Fourth Annual Session

**Published:** 1904-10

**Authors:** 


### F. D. I. INTERNATIONAL DENTAL FEDERATION—FOURTH ANNUAL SESSION.

The International Dental Federation convened on Friday, August 26, 1904, in Music Hall, Coliseum Building, St. Louis, Mo.

The meeting was called to order by the President, Dr. Charles Godon, Paris.

He introduced Dr. William Conrad, St. Louis, who delivered the welcoming address on behalf of the dental profession of the State of Missouri and of the city of St. Louis. To his hospitable and fraternal remarks, the chairman, Dr. Godon, responded as follows:

Members of the International Dental Federation, ladies, and gentlemen, the first thought I wish to express here on behalf of the International Dental Federation is one of admiration for your great nation, your mighty republic, to which are bound by so many ties of sympathy and gratitude all citizens, the admirers of liberty, progress, and civilization. Your magnificent exhibition constitutes a new proof of the progressive genius of the American nation and deserves a leading place among the accomplishments of human activity. We acknowledge a debt of gratitude to the American dental profession, to which modern dentistry owes so much, and to its eminent representative members who are here with us.

Hail to the members of the dental profession throughout the world who so kindly responded to our invitation, and who are now with us as members of the Executive Council. While speaking of these devoted fellow-workers in this great international task, let us not forget those who, because of unavoidable circumstances, are prevented from being with us at this time, likewise those who have left us forever.

To-day we inaugurate in St. Louis the session that finishes the work of the first period of the F. D. I. We shall soon have to report to the Fourth International Dental Congress upon the mission intrusted to us by the Third International Dental Congress, and will turn over to them our powers.

I shall not attempt to make a long report of the work of our body during that period of four years. That is the duty of our general secretary and devoted co-laborer, Dr. E. Sauvez, who has from the beginning worked indefatigably for the benefit of the cause. I only wish to recall to your minds the principal phases through which the F. D. I. has passed in its first period of existence. It originated in Paris, in 1900, through the enthusiasm of the twelve hundred congressists from every corner of the globe. It uttered its first lispings in Cambridge the following year—in that old English university, under the presidency of the eminent professor of physiology, Sir Michael Foster, who then determined the real philosophy of education as related to dental training, defining and limiting the sphere of influence of the F. D. I. in order to make of it a great international advisory council of dentistry. The next meeting took place in Stockholm, where, in the midst of discussions which had in view the planning of an international dental curriculum and the determining of the best conditions under which public dental services should be carried out for the greatest and most effective benefits to suffering humanity, it was decided that it should be in St. Louis that the Fourth International Dental Congress should be held. From that decision ensued a long, feverish, and fruitful contention about the interpretation to be given to the powers of the Federation or its representative members, and about the conditions of its participation in different kinds of professional movements. That discussion lasted over two years, and the session of Madrid in 1903 was almost exclusively devoted to it.

I have said feverish, inasmuch as it has caused deep discussions in our profession, especially in the United States, and fruitful also, because thus its existence has been made manifest to every one, and its importance as a factor in the proceedings of professional nature has been made equally evident.

We have therefore been enabled to prepare a constitution and a set of by-laws, which, when confirmed by the next Congress, will assure, I hope, the success of its future work.

In this, the twentieth century, these international federations constitute a new organism necessary not only to dentistry, but to all departments of professional and scientific activity. When men thus meet in great peaceful sessions and universal expositions and congresses, wholly given up to the pleasurê of exchanging ideas, and of communicating the result of their works and investigations, they must forget political and geographical limits and think only of the greatest good that may be derived for the benefit of all, regardless of nationality or local susceptibilities.

As new agents in the development of progress in civilization these great federations do not interfere with national questions, for those remain under the safe keeping of political and diplomatic bodies; but their activities take place on the high plane of human science, where territorial divisions have no action, where the end can be only peaceful because the contests are only of the intellect, and where the presiding goddess is like the statue of Liberty in the harbor of New York, having in one hand the torch of truth, and in the other the olive branch—both symbols of true human fraternity.

The President then called on Dr. C. C. Chittenden, Madison, Wis., president of the National Dental Association, who in a few appropriate words expressed his admiration for the work and purposes of the F. D. I.

Dr. H. A. Smith, Cincinnati, Ohio, was the next speaker. He made a short address, in which he referred to the far-reaching influence of the Federation's work and to his pleasure and satisfaction that such an eminent body of men should meet upon American soil.

Dr. Godon then called on Dr. Sauvez, the Secretary-General, who read an able and carefully prepared report, in which he reviewed the work of the F. D. I. from its organization to the present time.

After the reading of the Secretary's annual report, addresses were made by Dr. José J. Rojo, Mexico; Dr. Vincenzo Guerini, Italy; Dr. Florestan Aguilar, Spain; Dr. Alfred Burne, Australia; Dr. John Grevers, Holland; Dr. L. C. Bryan, Switzerland; Dr. N. S. Jenkins, Germany; Dr. R. B. Weiser, Austria; Dr. J. Y. Crawford, Nashville, Tenn.; Dr. J. D. Patterson, Kansas City, Mo.; Dr. H. B. Tileston, Louisville, Ky., and Dr. J. J. Reid, president of the National Association of Dental Examiners.

In the afternoon the several commissions which together constitute the International Dental Federation held separate sessions in the same building. The deliberations of the International Commission of Education were presided over by Dr. Truman W. Brophy, Chicago; those of the International Commission of Hygiene and Public Dental Service by Dr. N. S. Jenkins, Dresden, Germany; those of the Commission on International Dental Press by Dr. A. W. Harlan, New York, in the absence of the regular chairman, Dr. Elof Förberg, Stockholm, Sweden.

At the afternoon sessions and at those held on the following day papers were presented and read before the respective commissions,—by Dr. Charles Godon, on "Dental Education in France;" by Dr. William Mitchell, of London, on "Technical Education;" by Dr. W. E. Boardman, Boston, on "The Necessity for Establishing Libraries in Dental Schools;" by Dr. C. N. Johnson, Chicago, Ill., "A Brief Consideration of the Grading of Students in Dental Colleges;" by Dr. H. L. Banzhaf, Milwaukee, Wis., on "International Dental Education;" by Dr. A. H. Thompson, Topeka, Kan., on "The Development of Dental Education in the West;" by Dr. Gordon White, Nashville, Tenn., on "The Present Status of Dental Education in the United States;" by Dr. R. B. Weiser, Vienna, Austria, on "Education;" by Dr. Charles Godon, on "Emergency Dentistry and Complete Dentistry for the Poor;" by Dr. Burton Lee Thorpe, St. Louis, Mo., on "The *American Journal of Dental Science* and its Influence;" and by Dr. J. Endelman, Philadelphia, Pa., on the "International Federation Bulletin."

The Executive Council held several business meetings, and at the last one, held in the Coliseum Building on Saturday, September 3, prior to the closing of the Congress the following officers were elected:

Honorary President, Charles Godon, Paris; President, W. D. Miller, Berlin; First Vice-President, Emile Sauvez, Paris; Second Vice-President, R. B. Weiser, Vienna; Third Vice-President (to be elected by the British Dental Association); Secretary-General, Edward C. Kirk, Philadelphia; Assistant Secretaries, Shaffer-Stuckert, Frankfort, Paul Guye, Geneva, and Burton Lee Thorpe, St. Louis; Treasurer, Florestan Aguilar, Madrid.

The present Executive Council is composed of fifty members, each country affiliated with the Federation being represented by a number of members varying from one as the minimum to five as the maximum.

The present representatives of the United States, five in number, were elected by the National Dental Association at its meeting held in the Coliseum Building, September 2. They are William Carr, New York; B. Holly Smith, Baltimore; Edward C. Kirk, Philadelphia; A. W. Harlan, New York; Burton Lee Thorpe, St. Louis.

The next meeting of the Federation will be held in Switzerland in 1905, the date and the city to be announced in the near future.

RULES AND REGULATIONS OF THE INTERNATIONAL DENTAL FEDERATION, AS APPROVED BY THE FOURTH INTERNATIONAL DENTAL CONGRESS, AT ST. LOUIS, MO.

*Preamble.*

(a) The International Dental Federation is an association or universal union of national dental societies and those affiliated therewith.

(b) The official title adopted is "Fédèration Dentaire Internationale," abridged "F. D. I."

(c) The International Dental Federation is a permanent international body existing in the interim between international dental congresses.

(d) It is governed by an Executive Council, composed of delegates representing different countries (receiving appointment from the preceding congress). This Council organizes various Commissions that it deems will be beneficial to the advancement of dental science in any of its phases; it is at the same time an advisory committee on international affairs.

(e) The F. D. I. will hold a general meeting preceding the opening of each international dental congress.

(f) The Executive Council and the various Commissions will hold annual meetings, the time and place to be selected at the close of each meeting.

(g) *Authority creating the F. D. I.:* Resolutions passed by the Third International Dental Congress (Paris, France), August 14, 1900,—viz.:

"*Resolution 11.* There shall be organized an International Dental Federation.

"*Resolution 12.* The national committees appointed to this Congress will continue in office and will constitute the International Dental Federation."

*Rules and Regulations.*

ARTICLE I.—The International Dental Federation was organized by the national committees present at the Third International Dental Congress, at Paris, in 1900, and was created in conformity with Resolutions 11 and 12 passed by the general meeting on the closing day of the aforesaid Congress, August 14, 1900.

Art. II.—The objects of the Federation are as follows:

(a) The acceptance or rejection of invitations made by various countries to hold a regular International Dental Congress, and to fix the date and place where such congress shall be held.

(b) To maintain and strengthen the ties that bind the National Societies to each other.

(c) The organization of such International Commissions as it may deem necessary to create.

(d) In a general way, to promote the organization of bodies that will contribute to the advancement of odontological science throughout the world.

Art. III.—The International Dental Federation consists of—

(a) All the national committees gathered in Paris in 1900, or their successors.

(b) Associations or societies giving their adhesion to international dental congresses, and accepting these Rules and Regulations or sending their concurrence in them.

(c) Societies, or groups of societies, which may officially signify their acquiescence in these Rules and Regulations, and which are acceptable to the Executive Council.

Art. IV.—National dental associations or societies, or, in the absence of such, persons desiring to become identified with the F. D. I., should send their acceptance of the present Rules and Regulations. Such applications will be acted upon by the Executive Council, who will accept them as members of the Federation.

Art. V.—The general meeting of the F. D. I. will take place before the opening of each International Dental Congress. It will be composed of delegates from national or other societies. Extraordinary meetings may be called for special reasons by the Executive Council.

Art. VI.—The Executive Council may admit as members of the Federation—

(1) Members regularly appointed by societies.

(2) Honorary members.

(3) Persons in good professional standing who have been members of international dental congresses, and who shall subscribe to these Rules and Regulations.

Art. VII.—The programme for these meetings will be prepared by the Executive Council. It will deal with matters emanating from national or other societies, or with questions proposed by the

Council. Notices will be sent at least one month before these meetings to all affiliated societies, national or local.

Art. VIII.—The right of voting pertains to the regularly appointed delegates. Upon the request of representatives of at least two national dental associations, the vote may be taken by the said regularly appointed delegates in the mode of one vote for each country.

Art. IX.—The annual meetings of the Executive Council, and of the various commissions, are governed by the preceding Rules and Regulations.

Art. X.—The F. D. I. is composed of an Executive Council, as follows:

(1) Fifty original members, chosen by the Congress,—that is to say, for each country as a minimum one member, with a maximum of five members.

(2) In case of vacancy, by resignation, death, or other cause, the Council will ask the respective national dental association to fill the place of the missing member.

(3) The powers of the Executive Council will expire upon the opening of each International Dental Congress.

(4) The Council will hand over to a special committee appointed by the Congress all of its documents and records, at the time of opening of the Congress, the said committee receipting for the same.

(5) The treasurer of the F. D. I. will hold office until his successor is appointed.

Art. XI.—The Council is governed by nine officers, as follows:

(1) A president.

(2) Three vice-presidents.

(3) A secretary-general.

(4) Three assistant secretaries.

(5) A treasurer.

The officers of the Council are *ex officio* members of all commissions, and will direct them until they are properly organized.

Art. XII.—The duty of the Executive Council is—

(a) To supervise the execution of the rules of the Federation.

(b) To fix the place and date of annual meetings, and of International Dental Congresses.

(c) To organize various International Commissions.

(d) To supervise the carrying out of decisions made by the F. D. I.

(e) To examine propositions and resolutions offered by national committees, associations, or other societies.

The Council will keep all affiliated bodies informed of their work through the Bulletin of the Executive Council, which will be published in at least four languages,—viz., French, German, English, and Spanish.

Art. XIII.—The Council has already named several special Commissions, as follows:

(1) A Commission on Education.

(2) A Commission on Hygiene and Public Dental Service.

(3) A Commission on International Dental Press.

And it will organize a Commission on Professional Jurisprudence, Deontology, and Nomenclature.

Art. XIV.—The sources of income of the F. D. I. are as follows:

(1) By dues from the members,—to wit: Members of the Executive Council (per year), $10.00; members of Commissions (per year), $5.00; honorary members and all others (per year), $5.00.

(2) Appropriations by Congresses.

(3) Subscriptions, gifts from governments or municipalities, from national associations, and from individuals.

Art. XV.—In case of deficit, the expenses of the F. D. I. shall be provided for by equal assessment on all societies having membership. Any excess above the receipts will be turned over to the next Dental Congress. The Council will give a detailed statement of receipts and expenditures to every Congress.

Art. XVI.—The Executive Council will send to the Congress during its sessions a list of those members best qualified to carry on the international work of the F. D. I.

Art. XVII.—

(1) International Dental Congresses shall be organized by a committee, composed of dentists, who shall be chosen as follows:

One-third of its membership shall be appointed by the Executive Council of the F. D. I.; the other two-thirds shall be appointed by the inviting dental bodies. The committee so composed shall constitute the Committee of Organization, all the members of which shall have the same powers.

(2) At the first meeting of the Committee of Organization they shall organize and select the following officers of the committee: One president; two vice-presidents; one secretary-general; one treasurer.

(3) The Executive Council of the F. D. I. has full power to decide all questions in dispute arising in the Committee of Organization.

ART. XVIII.—These Rules are operative during the periods between regular Congresses. They are subject to revision by the succeeding Congress.

